# Population genetics of *Enterocytozoon bieneusi* in captive giant pandas of China

**DOI:** 10.1186/s13071-017-2459-z

**Published:** 2017-10-18

**Authors:** Wei Li, Yuan Song, Zhijun Zhong, Xiangming Huang, Chengdong Wang, Caiwu Li, Haidi Yang, Haifeng Liu, Zhihua Ren, Jingchao Lan, Kongju Wu, Guangneng Peng

**Affiliations:** 1 The Key Laboratory of Animal Disease and Human Health of Sichuan Province, College of Veterinary Medicine, Sichuan Agricultural University, Sichuan Province, China; 2 Sichuan Key Laboratory of Conservation Biology for Endangered Wildlife, Chengdu Research Base of Giant Panda Breeding, Chengdu, Sichuan Province China; 3 Wolong Giant Panda Base, Aba, Sichuan Province China

**Keywords:** *E. bieneusi*, Multilocus sequence typing, Gene diversity, Population genetics, Population structure, Linkage disequilibrium

## Abstract

**Background:**

Most studies on *Enterocytozoon bieneusi* are conducted based on the internal transcribed spacer (ITS) region of the rRNA gene, whereas some have examined *E. bieneusi* population structures. Currently, the population genetics of this pathogen in giant panda remains unknown. The objective of this study was to determine the *E. bieneusi* population in captive giant pandas in China.

**Results:**

We examined 69 *E. bieneusi*-positive specimens from captive giant pandas in China using five loci (ITS, MS1, MS3, MS4 and MS7) to infer *E. bieneusi* population genetics. For multilocus genotype (MLG) analysis of *E. bieneusi*-positive isolates, the MS1, MS3, MS4, and MS7 microsatellite and minisatellite loci were amplified and sequenced in 48, 45, 50 and 47 specimens, respectively, generating ten, eight, nine and five types. We successfully amplified 36 specimens and sequenced all five loci, forming 24 MLGs. Multilocus sequence analysis revealed a strong and significant linkage disequilibrium (LD), indicating a clonal population. This result was further supported by measurements of pairwise intergenic LD and a standardized index of association (*I* ^S^_A_) from allelic profile data. The analysis in STRUCTURE suggested three subpopulations in *E. bieneusi*, further confirmed using right’s fixation index (*F*_ST_). Subpopulations 1 and 2 exhibited an epidemic structure, whereas subpopulation 3 had a clonal structure.

**Conclusions:**

Our results describe *E. bieneusi* population genetics in giant pandas for the first time, improving the current understanding *E. bieneusi* epidemiology in the studied region. These data also benefit future studies exploring potential transmission risks from pandas to other animals, including humans.

## Background

Diarrheal disease is the second leading cause of morbidity and mortality in children under 5 years old (including infants) in low-income countries [1, 2]. The illness can often be traced to the unicellular fungus microsporidian, recognized as a category B biodefense agent on the National Institutes of Health list [3]. *Enterocytozoon bieneusi* is among the most common microsporidia; in immunocompetent individuals, the anthropozoonotic pathogen may be asymptomatic, but it has been implicated in self-limiting diarrhoea and malnutrition. In immunosuppressed individuals, however, persistent diarrhoea, abdominal pain, and weight loss are common symptoms [3–5]. *Enterocytozoon bieneusi* can also infect various invertebrates and vertebrates, including domestic animals, through a wide variety of transmission routes: water-borne, food-borne, anthroponotic and zoonotic [6, 7]. Currently, effective commercial treatments for *E. bieneusi* infection do not exist, although some reports have suggested that the fungicide fumagillin is effective. However, this drug is toxic to mammals [6].

*Enterocytozoon bieneusi* cannot be identified based on morphology because its spores are too small and do not have characteristic staining properties, making them indistinguishable. Moreover, in vitro cultivation of the fungus has been unsuccessful thus far [8, 9]. Thus, molecular screening is currently the only option. ITS gene analysis is widely used in molecular epidemiological studies of genetically diverse *E. bieneusi*, identifying over 200 genotypes [10, 11]. ITS analysis is adequate for genotyping and epidemiological studies of *E. bieneusi* in the absence of recombination. However, ITS analysis has major limitations if genetic recombination occurs at any reproduction stage [12]. As a result, multilocus genotyping (MLST) has been developed, targeting three microsatellites (MS1, MS3 and MS7) and a minisatellite (MS4) to provide high-resolution *E. bieneusi* genotyping based on length polymorphisms and single nucleotide polymorphisms (SNP) [13]. Together with ITS analysis, multilocus genotyping has been gradually applied to *E. bieneusi* genetic characterization. The results contribute to the establishment of effective control measures by enabling elucidation of transmission routes, characterization of the public health consequences of animal-derived *E. bieneusi* isolates, and identification of the sources of human *E. bieneusi* infections.

For *E. bieneusi*, MLST has been applied to humans, non-human primates, swine, Asiatic black bears, red kangaroos, dairy and native beef, foxes, and red-bellied tree squirrels [14–21]. However, no population genetic analysis is available on *E. bieneusi* in giant pandas. Therefore, this study aimed to amplify the 12 ITS sequences in *E. bieneusi*-positive isolates from captive giant pandas of China using micro/minisatellite markers and characterize them using MLST. The resultant data on population genetic structure will help to prevent or reduce microsporidiosis occurrence in giant pandas through the development of efficient control strategies. These data can also be used to compare *E. bieneusi* population genetics across multiple hosts, improving risk assessment of potential human and livestock infection through infected giant pandas.

## Methods

### *Enterocytozoon bieneusi* isolates

In total, 69 ITS-positive *E. bieneusi* isolates from captive giant pandas were previously genotyped in our laboratory. Samples were collected from captive giant pandas at conservation bases and zoological gardens in china. Genotypes SC02 (*n* = 50), D (*n* = 3), SC06 (*n* = 2), CHB1 (*n* = 2), F (*n* = 2), EbpC (*n* = 2), SC01 (*n* = 2), SC04 (*n* = 2), SC05 (*n* = 1), SC07 (*n* = 1), SC08 (*n* = 1) and Peru 6 (*n* = 1) were used for MLST analysis.

### MLST PCR and sequencing

Three microsatellites (MS1, MS3 and MS7) and one minisatellite (MS4) were further amplified in all 69 samples to determine their multilocus genotypes (MLGs). Primers and amplification conditions were described previously [13]. Secondary PCR products were visualized by Golden View staining and 1% agarose gel electrophoresis. Amplicons of the expected size (MS1, 676 bp; MS3, 537 bp; MS4, 885 bp; MS7, 471 bp) were bi-directionally sequenced by Invitrogen (Shanghai, China) to ensure accuracy.

### Genetic analysis

We analyzed *E. bieneusi* genetic diversity at each marker (ITS, MS1, MS3, MS4 and MS7) in DnaSP version 5.10.1 (http://www.ub.edu/dnasp/). Detailed methods for calculating genotype frequency, genetic diversity (Hd), and intragenic linkage disequilibrium (LD) followed a previous publication [14]. We then concatenated sequences of *E. bieneusi* isolates for all five loci separately. Sequence alignment determined MLGs. DnasP and Arlequin version 3.5.2.2 (http://cmpg.unibe.ch/software/arlequin35/) were used to calculate nucleotide diversity (Pi), genotype frequency, Hd, LD, and recombination rates. To determine the nature of mutations in the population, Fu’s neutrality tests (*F*s) were conducted in both programs based on segregating sites (DnaSP), as well as on segregating sites and indels (Arlequin). Significant differences were assessed with coalescence simulations in a parameter test. To confirm the occurrence of recombination, rates were estimated using GENECONV, MaxChi, and SiScan in RDP (Recombination Detection Program) version 4.92 (http://web.cbio.uct.ac.za/~darren/rdp.html).

A population genetic analysis of allelic profiles was also performed. In Arlequin, we applied exact tests with Markov chain parameters to allelic profile data to assess pairwise intergenic LD. Standardized index of association (*I* ^S^_A_) values were calculated using LIAN 3.7 (http://guanine.evolbio.mpg.de/cgi-bin/lian/lian.cgi.pl) on five-loci haplotypes. *I* ^S^_A_ equal to zero or with a negative value indicates randomly mating populations and alleles in linkage equilibrium (LE). An *I* ^S^_A_ value greater than zero indicates a non-panmictic population structure is exhibiting LD. The variance of pairwise differences (V_D_) was also calculated as another test of LD; when less than L (95% critical value for V_D_ relative to the null hypothesis of panmixia), the population is panmictic and is in LE. When V_D_ > L, the population is non-panmictic and has some LD [22]. Intra-/intergenic LD, *I* ^S^_A_, neutrality, and recombination events were combined to assess *E. bieneusi* population structure.

### Substructure analysis

Allelic profile data were analyzed to determine subpopulations of *E. bieneusi* genotypes, using K-means partitional clustering and the admixture model in the Bayesian analysis tool STRUCTURE version 2.3.4 (http://web.stanford.edu/group/pritchardlab/structure.html) [23]. Twenty simulation runs were conducted for each of *K* value = 2–8, with 10^4^ and 10^5^ replicates of the Markov chain Monte Carlo simulation discarded as burn-in. Wright’s fixation index (*F*_ST_), a measure of population divergence, was calculated in Arlequin; *F*_ST_ = 0 indicates similar polymorphisms across all markers and *F*_ST_ = 1 indicates high levels of between-population divergence. We also performed a median-joining analysis implemented in the software Network version 5.0 (http://www.fluxus-engineering.com/sharenet.htm) to estimate the potential for geographic segregation.

## Results and discussion

Among the 69 ITS-positive *E. bieneusi* isolates, the MS1, MS3, MS4 and MS7 loci were successfully amplified in 48, 45, 50 and 47 specimens, respectively, yielding ten, eight, nine, and five types (Table 1). MS1 exhibited the highest sequence polymorphism and MS7 the lowest. A previous study of *E. bieneusi* population genetics in humans reported the highest resolution for MS1 and the lowest for MS3 and MS4 [14]. Another study conducted in non-human primates also suggested that MS1 had the highest resolution and MS3 the lowest [16]. In contrast, research on *E. bieneusi* populations in swine revealed that MS3 and MS4 possessed higher resolution than the other tested loci [15]. These inter-study differences may result from variation in hosts and sample size. The Hd and intragenic linkage disequilibrium shown in Table 2 indicate that all five markers were in significant LD. Assessment of intragenic recombination revealed that ITS and MS4 exhibited one and two recombination events (Rms), respectively, and LD was incomplete in these two loci, whereas the remaining three markers (with complete LD) did not have Rms (Table 2).

**Table 1:** Samples used in this study and their genotype distribution

Category	No. of samples used	No. of types at each micro and mini satellite loci^a^				No. MLGs
		MS1	MS3	MS4	MS7	
CRBGPB	25	7 (15)	5 (14)	4 (15)	4 (14)	10 (11)
DGPB	16	5 (14)	2 (12)	5 (14)	2 (14)	7 (11)
WGGPB	7	1 (6)	4 (5)	2 (6)	1 (6)	3 (4)
WHGPB	3	1 (1)	2 (2)	2 (2)	0 (0)	0 (0)
YBGPB	4	2 (3)	3 (3)	3 (3)	1 (3)	3 (3)
ZGs	14	4 (9)	2 (9)	6 (10)	1 (10)	6 (7)
Total	69	10 (48)	8 (45)	9 (50)	5 (47)	24 (36)

^a^Number in the parenthesis indicates the number of samples

*Abbreviations: CRBGPB* Chengdu Research Base of Giant Panda Breeding, *DGPB* Dujiangyan Giant Panda Base, *WGGPB* Wolong Gengda Giant Panda Base, *WHGPB* Wolong Hetaoping Giant Panda Base, *YBGPB* Yaan Bifengxia Giant Panda Base, *ZGs* zoological gardens

**Table 2:** Hd, polymorphic sites, intragenic linkage disequilibrium and recombination events in individual genetic markers among 36 *E. bieneusi* specimens

Marker	Hd	Polymorphic sites	LD (|D’|)	Rm
ITS	1	43	Y = 0.9994 + 0.0000X	1
MS1	0.53	6	Y = 1.0000 + 0.0000X	0
MS3	0.86	7	Y = 1.0000 + 0.0000X	0
MS4	0.97	28	Y = 0.9866 + 0.0163X	2
MS7	1	14	Y = 1.0000 + 0.0000X	0

*Hd* gene diversity, *LD* (|D’|) linkage disequilibrium per site, *Rm* minimum number of recombination events

All five loci were amplified and sequenced for 36 specimens, forming 24 MLGs with an Hd of 0.77 (Table 1). The 36 contigs had 49 polymorphic sites, with nucleotide diversity Pi = 0.00439 and an average number of nucleotide differences k = 8.379. In this study, the 36 isolates yielded 24 MLGs with a Hd value of 0.994 or 0.768, depending on whether the analysis used polymorphic or segregating sites. The results indicate that *E. bieneusi* genotypes are relatively abundant in our giant panda population. However, nucleotide diversity was very low (0.033 based on polymorphic sites, 0.004 based on segregating sites). This pattern of high Hd but low nucleotide diversity suggests the presence of a bottleneck followed by population expansion in *E. bieneusi* infecting giant pandas.

**Fig. 1:**
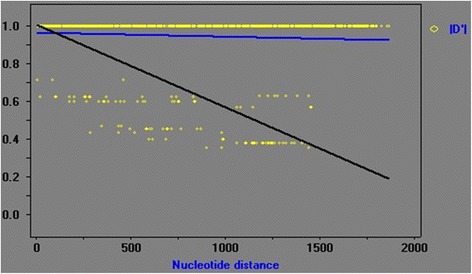
Linkage disequilibrium (LD) in 36 isolates determined by DnaSP software after end-to-end linking of the sequences of 5 loci. Y coordinate represents the LD value, and X coordinate represents the nucleotide distance in kb

The observed ZnS, a measure of LD, was 0.254. Fisher’s exact test found 339 significant pairwise correlations out of 1176, with 111 remaining significant after a Bonferroni correction. In contrast, chi-squared tests found 605 significant correlations, with 300 remaining significant after Bonferroni corrections. Coalescence simulations with 1000 replicates showed that average ZnS was 0.120, and its 95% confidence interval ranged from 0.066 to 0.462. The probability for expected ZnS ≤ 0.254 was 0.200 (i.e. the observed ZnS had a 0.200 probability of being higher than the expected ZnS if the former was within the 95% confidence interval). Linkage disequilibrium was strong and significant (|D’|: Y = 0.9652 - 0.0204X), but a negative slope in the |D’| graph (Fig. 1) revealed a decline in an LD with greater nucleotide distance, leading to the possibility of genetic recombination. Accordingly, we also detected a minimum of four Rms, but we note that they could be due to genetic changes. To further confirm the occurrence of recombination, we utilized GENECONV, SiScan, and MaxChi analyses implemented in RDP 4 software. SiScan analysis did not detect any Rm, whereas both GENECONV and MaxChi detected two Rms (all three tests ignored indels).

The results of Fu’s neutrality test shown in Table 3 indicate that the population experienced molecular selection. Moreover, the observation of Fs = -6.680 (the infinite model of mutation) suggested that a great number of alleles were present that probably resulted from a recent population expansion.

**Table 3:** Genetic diversity in 36 *E. bieneusi* specimens based on the analysis of concatenated multilocus gene sequences

Test model	Variability of multilocus gene sequences						
	No. of MLGs	Hd	k	Pi	Theta (k)	*F*s (Obs)	*P* (*F*s ≤ Obs)
Polymorphic sites	662	0.994	83.173	0.0330	83.173	-6.680	0.010
Segregating sites	49	0.768	8.379	0.004	8.379	1.144	0.133

*Abbreviations: Hd* gene diversity, *k* mean number of pairwise differences, *Pi* nucleotide diversity (average over loci), *theta (k)* gene variance based on the mean number of pair-wise differences, *Fs* Fu’s statistic testing selective neutrality based on genotype frequency, *Obs* observed value, *P* (Fs ≤ Obs) probability of obtaining *F*s values equal or lower than the observed

We found an *I* ^S^_A_ of 0.0903 and a V_D_ (0.9725) greater than L (0.7652) (Table 4), indicating a non-panmictic population exhibiting LD. Furthermore, our Monte Carlo simulations revealed a P_MC_ (significance for obtaining this value in 1000 permutations) below the threshold for retaining the null hypothesis of panmixia (Table 4). Thus, all three tests indicated a non-panmictic population is exhibiting LD. To exclude the possibility that the LD was due to clonal expansion of one or more MLGs, we also calculated *I* ^S^_A_ and V_D_ for MLGs another way, considering isolates with the same MLG as a single individual. The *I*_A_^S^ value was 0.0408 and V_D_ value was still higher than L (Table 4). Thus, the population of giant pandas in our study was clonal with strong LD and limited recombination, which suggests that MLGs are relatively stable across time and location. Thus, they should be used for tracking the transmission of microsporidiosis.

**Table 4:** Results of linkage disequilibrium analysis based on allelic profile data

Population	*n*	H ± SD	*I* ^S^_A_	P_MC_	V_D_	L	V_D_ > L
All	36	0.5151 ± 0.1396	0.0903	1.10 × 10^−02^	0.9725	0.7652	Y
All^a^	23	0.6107 ± 0.1358	0.0408	2.11 × 10^−01^	0.8191	0.7297	Y
Population 1	14	0.2637 ± 0.1539	0.0482	1.97 × 10^−01^	0.5636	0.4925	Y
Population 2	10	0.2833 ± 0.1641	-0.0358	–	0.4364	0.4889	N
Population 3	12	0.6856 ± 0.0908	0.0739	1.76 × 10^−01^	0.9326	0.7633	Y
Population 1^a^	7	0.5833 ± 0.1463	-0.0331	7.75 × 10^−01^	0.6443	0.7153	N
Population 2^a^	6	0.3167 ± 0.1873	-0.0690	–	0.3524	0.4444	N
Population 3^a^	12	0.6856 ± 0.0908	0.0739	1.76 × 10^−01^	0.9326	0.7633	Y

^a^Considering each group of isolates with the same MLG as one individual

*Abbreviations: n* number of isolates, *H* mean genetic diversity, *SD* standard deviation, *I* ^*S*^_*A*_ standardized index of association, *P*_*MC*_ significance for obtaining this value in 1000 simulations using the Monte Carlo method, *V*_*D*_ variance of pair-wise differences, *L* 95% critical value for V_D_, V_D_ > L indicates linkage disequilibrium, *Y, V*_*D*_ *> L* N, V_D_ ≤ L

**Fig. 2:**
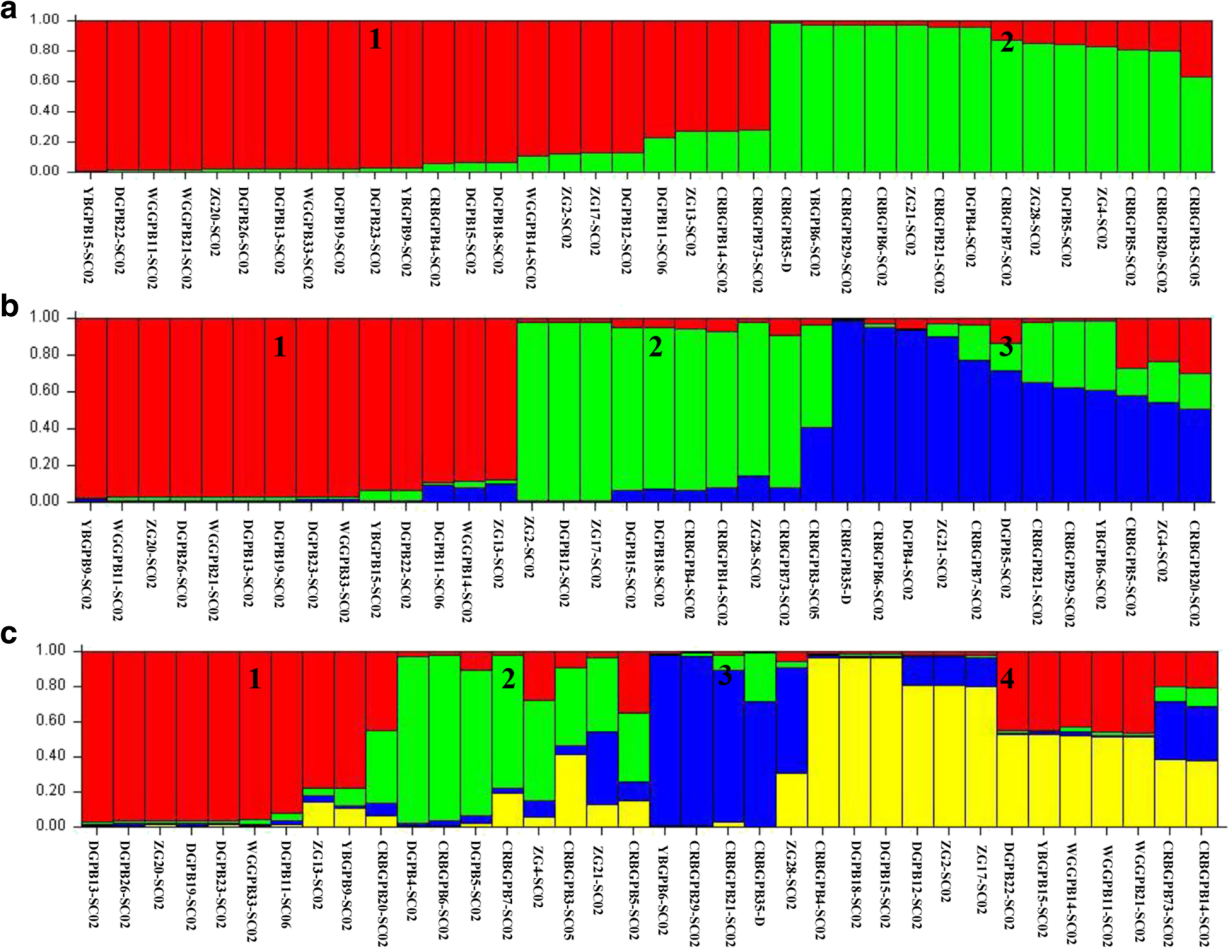
Subpopulation structure of 36 *E. bieneusi* isolates. Different subpopulation patterns are shown depending on the setting of K values. **a** K = 2. **b** K = 3. **c** K = 4

**Fig. 3:**
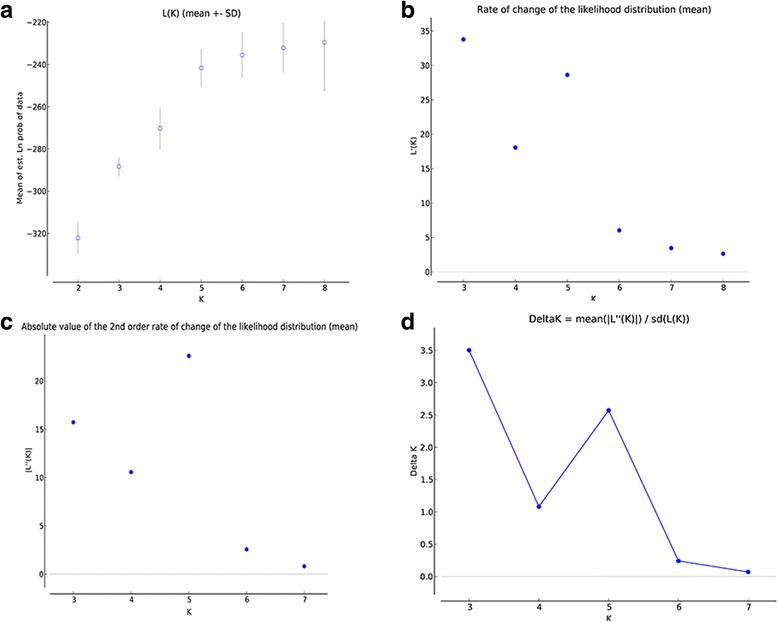
K value calculation for population substructuring after burn-in length of 10^4^ and 10^5^ replicates of 20 Markov chain Monte Carlo simulation runs. **a** Plot of mean likelihood L (K) and variance per K value from STRUCTURE on a dataset containing 36 specimens for five loci. **b-d** Plots for detecting the number of K groups that best fit the data, where (**d**) represents the peak delta K value of 3

A STRUCTURE analysis was used to further assess the formation of subpopulations. As shown in Fig. 2, when K = 2 (Fig. 2a) and 3 (Fig. 2b), the subpopulations generated were clear, whereas a staggered pattern was formed when K = 4 (Fig. 2c). α values remained stable at < 0.2 when K = 2 (α = 0.0886), 3 (α = 0.0432) and 4 (α = 0.0424), which demonstrates the presence of subpopulations [14]. The presence of three subpopulations was also confirmed with 20 Monte Carlo simulation runs, which showed a peak value of Delta K at K = 3 (Fig. 3). Furthermore, two subpopulations (K = 2) exhibited *F*_ST_ = 0.230, suggesting extensive genetic differences. Subpopulations were further confirmed with comparisons of *F*_ST_ across any two subpopulations (K = 3), which yielded *F*_ST_ values (0.066, 0.168, and 0.127) consistently over 0.05.

The LD analysis of the allelic profiles from subpopulations 1, 2 and 3 revealed positive *I* ^S^_A_ for subpopulation 1 and 3, along with and V_D_ > L with insignificant P_MC_. However, subpopulation 2 had negative *I* ^S^_A_ and V_D_ < L (Table 4). To confirm the existence of epidemic population structure, we then recalculated *I* ^S^_A_ for MLGs (isolates with the same MLG were treated as one individual) of the three subpopulations. Subpopulations 1 and 2 had negative *I* ^S^_A_ and V_D_ < L with insignificant P_MC_, whereas subpopulation 3 had positive *I* ^S^_A_ and V_D_ > L. Thus, both subpopulations 1 and 2 were in LE and had epidemic population structure, whereas subpopulation 3 remained in LD (despite non-significant P_MC_), exhibiting a clonal population structure. This outcome was similar to a previous study in AIDS patients, showing one *E. bieneusi* subpopulation with a clonal structure and another with an epidemic population structure [24]. A clonal population structure of *E. bieneusi* was also found in non-human primates and swine [15, 16].

**Fig. 4:**
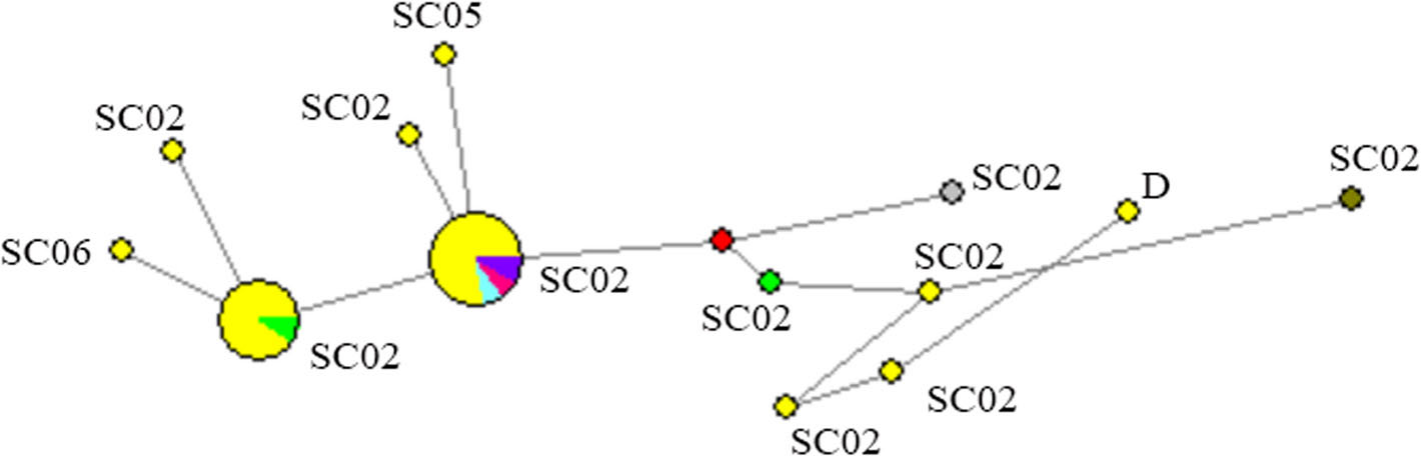
Median-joining analysis of the multilocus sequence contigs from 36 *E. bieneusi* isolates in this study. The size of the circles is proportional to the frequency of each of the 24 multilocus genotypes obtained based on segregating sites. The yellow, green, pink, purple, blue, grey and brown colours in circles represent the isolates from Sichuan, Zhejiang, Guangdong, Shandong, Fujian, Hunan provinces, and Shanghai city, respectively

All the clusters formed mainly came from Sichuan province in the substructure analysis (Fig. 2b). Thus, no clear geographically segregated subpopulation was observed. While, subpopulation structure analysis showed that subpopulation 1 consisted of isolates only from adults (> 5.5 years); subpopulation 2 had admixture of isolates from adults and yearlings (< 1.5 years), and adults isolates dominated; subpopulation 3 mainly consisted of isolates from juveniles (1.5–5.5 years) which may have contributed to the significance for LD and *I* ^S^_A_ analyses of each population. In the median-joining network analysis, all the clusters were all mainly from Sichuan province (Fig. 4). We still observed no clear geographical segregation of *E. bieneusi* isolates from different areas, which is similar to the report of Li et al. in 64 isolates from AIDS patients and captive baboons [24].

## Conclusions

We used ITS and micro/minisatellite markers to analyze 69 *E. bieneusi* isolates from captive giant pandas of China. We found an overall clonal population of *E. bieneusi* with strong LD and limited recombination. Within the larger population, three subpopulations were present, with two exhibiting epidemic structure (subpopulations 1 and 2), whereas the third exhibited clonal structure (subpopulation 3). Additional large-scale MLST studies on *E. bieneusi* in other hosts should be conducted to characterize the pathogen’s population genetics better. Such data should prove highly beneficial for characterizing *E. bieneusi* risk factors, including transmission, host specificity, and exact clinical presentation.

## Abbreviations

*F*s: Fu’s neutrality tests
*F*_ST_: Right’s fixation index
Hd: Genetic diversity
*I*^S^_A_: A standardized index of association
ITS: Internal transcribed spacer
LD: Linkage disequilibrium
LE: Linkage equilibrium
MLG: Multilocus genotype
MLGs: Multilocus genotypes
MLST: Multilocus sequence typing
Pi: Nucleotide diversity
RDP: Recombination detection program
SNPs: Single nucleotide polymorphisms
V_D_: Variance of pairwise differences; Rms: recombination events
